# Dietary Supplementation of Ethanolic Lemon Peel (*Citrus limon*) Extract Enhances Growth Performance and Immune-Antioxidant Responses in Pacific White Shrimp (*Litopenaeus vannamei*)

**DOI:** 10.1155/anu/4879504

**Published:** 2025-06-30

**Authors:** Hadis Eslami, Leila Abdoli, Arash Akbarzadeh

**Affiliations:** Department of Fisheries, Faculty of Marine Science and Technology, University of Hormozgan, Bandar Abbas, Iran

**Keywords:** antioxidant, gene, immune system, lemon, shrimp

## Abstract

Citrus (*Citrus limon*) by-products, particularly lemon peel, are rich in bioactive compounds with known antioxidant and anti-inflammatory properties. This study evaluated the effects of lemon peel extract (LPE) supplementation on growth performance, immune responses, and antioxidant activity in Pacific white shrimp (*Litopenaeus vannamei*). Over a 60-day period, shrimp were fed four diets: a control diet (0%) and three diets with varying levels of LPE (0.5%, 1%, and 2%). The results showed that shrimp fed the 1% LPE diet exhibited significantly higher weight gain (WG), specific growth rate (SGR), and lower feed conversion ratio (FCR) compared to the control group (*p* < 0.05). Additionally, shrimp fed 0.5% and 1% LPE showed significantly enhanced activities of antioxidant enzymes (catalase [CAT], glutathione peroxidase [GPX], superoxide dismutase [SOD]), lysozyme, total protein, and hemocyte counts, including large granular cells (GCs), semi-granulocytes, and hyaline cells (HCs) (*p* < 0.05). Total hemocyte counts (THCs) were also significantly higher in all LPE-treated groups compared to the control (*p* < 0.05). Plasma glucose levels were reduced in the 0.5% and 1% LPE treatments, though the decrease in cholesterol was not statistically significant. Moreover, the expression of *integrin β*, *PX*, and *α2-M* mRNA was significantly upregulated in shrimp fed the 2% LPE diet, while *LGBP* and *proPO1* mRNA expression were higher across all LPE treatments. In conclusion, dietary supplementation with LPE, particularly at 0.5% and 1%, enhances growth performance, immune response, and antioxidant activity in *L. vannamei*. Therefore, LPE presents a promising and cost-effective feed additive for shrimp aquaculture, with the potential to enhance disease resistance, support immune function, and ultimately improve shrimp health and productivity under farming conditions.

## 1. Introduction

Aquaculture has been growing at an annual rate of 2.8% over the past few decades [1]. In 2022, the global aquatic animal production reached ~185 million tonnes, with 94 million tonnes (51%) coming from aquaculture [2]. The global crustacean aquaculture production exceeded 12.8 million tonnes, with Pacific white shrimp (*Litopenaeus vannamei*) contributing significantly at 6.8 million tonnes [2]. This species is highly valued for its economic importance, high nutritional content, and appealing taste [3, 4].

Shrimp aquaculture has been severely impacted by deteriorating environmental conditions, including poor water quality, overcrowding, and inadequate farm management practices, all of which compromise the shrimp's immune system, making them more vulnerable to various infectious diseases, which have led to substantial economic losses in the industry [5, 6]. Frequent disease outbreaks, such as those caused by *Vibrio* bacteria and White Spot Syndrome Virus (WSSV), have decimated shrimp populations, threatening the sustainability of shrimp farming [7]. One of the most promising strategies to mitigate these risks is enhancing the innate immune system of farmed shrimp. Strengthening the shrimp's immune response through nutritional supplements, probiotics, and other management interventions has shown potential in boosting their resilience against diseases [8].

In recent years, plant products and by-products have been increasingly used as natural immune stimulants in fish and shellfish feed formulations due to their environmentally friendly properties and cost-effectiveness compared to synthetic drugs [9–11]. Citrus fruits, *Citrus limon* are among the most widely distributed and commercially cultivated fruits worldwide, with a significant presence in both tropical and subtropical regions. Known for their high vitamin C content, lemons are also rich in antioxidants, essential oils, and citric acid, which contribute to their health benefits, including boosting immunity and aiding digestion [12]. Although a significant part of the industrially processed citrus fruits is used to produce essential oils and juice, a large quantity of waste and by-products, including lemon peel and pomace, is yearly produced during citrus processing [13]. Citrus by-products are easy, accessible, and low-cost food supplements that can provide an efficient, inexpensive, and environmentally friendly basis for producing new food sources [14]. Studies have shown that lemon peel is rich in bioactive compounds that offer numerous benefits. It contains several active compounds, including dietary fibers (64.07 g/100 g DM), ascorbic acid (25.68 mg/100 g), flavonoids (59.9 mgCE/g), phenolic compounds (61.23 mgGAE/100 g), d-Limonene (0.7%–1.4%), linoleic acid (13.76%–36.9% oil), pectin (22.53%) and different vitamins and minerals [15–17]. Lemon peel extract (LPE) contains a mixture of over 400 known compounds, divided into two fractions: volatile (85%–90% of the total extract) and nonvolatile (10%–15%) [18]. One of the most important bioactive compounds in LPE is limonene, which has proven antibacterial, anti-inflammatory, antioxidant, and antiviral properties [19].

In aquaculture, citrus by-products have been supplemented into the diets of various fish species with promising results. Lemon peel supplementation improved the immune status and antioxidant activity of gilthead seabream, *Sparus aurata* [20]. In the diets of Nile tilapia, *Oreochromis niloticus* and African catfish, *Clarias gariepinus*, lemon peel enhanced enzymatic antioxidant capacity and immune response, although no significant effect on growth performance was observed [21]. It is also found that fermented LPE mitigated the stress effects of deltamethrin on growth performance, carcass composition, and antioxidant immune responses in rainbow trout, *Oncorhynchus mykiss* exposed to deltamethrin pesticide stress [22]. Furthermore, incorporating fermented lemon peel into the diet of Asian sea bass, *Lates calcarifer* [23] and orange-spotted grouper, *Epinephelus coioides* [24] improved intestinal health and the immune system. Recent research on shrimp has shown that *Lactobacillus plantarum*-fermented lemon peel can enhance the non-specific immune system of *L. vannamei* [25].

This study aimed to investigate the effectiveness of LPE in the diet of cultured shrimp *L. vannamei*, given its proven benefits and positive properties. We supplemented the shrimp diet with ethanolic extract of lemon peel at varying doses and assessed growth performance, survival, the hemolymph immune-antioxidant, and biochemical parameters of the shrimp. Additionally, we evaluated the mRNA expression of a panel of immune-related genes, including *α2-macroglobulin* (*α2-M*), *beta-1*,*3-glucan-binding protein* (*LGBP*), *integrin β*, *peroxinectin* (*PX*), and *prophenoloxidase* 1 (*proPO1*) in *L. vannamei* fed diets containing different amounts of LPE.

## 2. Materials and Methods

### 2.1. Diet Preparing

To prepare the LPE, the peels of *C. limon* were meticulously separated from the fruit, washed with water, and then dried in the shade at room temperature for a week. After drying, the peels were powdered using an electric mill. To obtain the extract, the powder was mixed with 70% ethanol alcohol at a volume ratio of 1:10 (weight/volume). The mixture was poured into an Erlenmeyer flask, covered with aluminum foil, and placed on a hot plate at room temperature for 72 h to ensure thorough mixing. The mixture was then filtered using Whatman filter paper and a glass funnel. The resulting liquid was placed in a rotary evaporator at 50°C to evaporate the alcohol. Finally, the concentrated extract was stored at 4°C until use [26, 27].

In this study, a commercial shrimp diet containing 42% protein, 9% lipid, 12% ash, 11% moisture, and 5% fiber was purchased from Hormozdam Aquatic Production and Trading Holding (Bandar Abbas, Iran), which served as the basal diet (control) with no inclusion of LPE. Subsequently, various concentrations of LPE were added to the basal diet to prepare the experimental diets, including 0.5%, 1%, and 2%. The selected doses of LPE were based on previous studies that incorporated LPE into the diets of fish species (e.g., [28, 29]). The LPE was solved in distilled water and subsequently sprayed onto the basal shrimp feed. The control diet was sprayed with an equal volume of distilled water to ensure consistency in feed moisture content and handling.

### 2.2. Experimental Design

Six hundred shrimp, initially weighing 4.0 ± 0.2 g, were divided into four groups: a control group receiving the basal diet without LPE and three experimental groups receiving diets with 0.5%, 0.1%, and 2% LPE. Within each treatment, 150 shrimp were randomly distributed across three 300 L circular fiberglass tanks, with three replicates (50 shrimp per tank). Following a 2-week acclimation period in the 300 L tanks and subsequent feeding with commercial shrimp feed, the shrimp were transitioned to the experimental diets for a period of 60 days. Daily water replacements were about 50%. The shrimp were fed with experimental diets at a rate of 5% body weight per day at 8:00, 14:00, and 20:00. Every 2 weeks, the daily feed amount was readjusted according to the total weight of shrimp in each replicate. Water conditions, including temperature (29 ± 2°C), oxygen levels (8.2 ± 0.5 mg L^−1^), pH (7.5), and salinity (35 ± 0.5 g L^−1^), were meticulously maintained. After feeding the shrimp with experimental diets for a period of 60 days, the shrimp were withheld from feeding for 24 h before being measured for growth parameters and sampled. Before sampling, shrimp were euthanized by thermal shock through immersion in iced saltwater maintained below 4°C. Subsequently, body weight and total length were recorded. Hemolymph and hepatopancreas samples were collected from three shrimp per tank for immune-antioxidant analyses and evaluation of immune-related gene expression, respectively. All procedures were conducted in full compliance with the ethical guidelines of the Iranian Society for the Prevention of Cruelty to Animals.

### 2.3. Growth Parameters

Growth parameters of shrimp, including weight gain (WG), total length increase (TLI), specific growth rate (SGR), and feed conversion rate (FCR), were measured as follows [30]:

  $$\begin{array}{c} WG\;\left(g\right)=W_{f}-W_{i}, \end{array}$$

  $$\begin{array}{c} TLI\;\left(mm\right)=\;L_{f}-L_{i}, \end{array}$$

  $$\begin{array}{c} SGR=100\times \left(L_{n}\;W_{f}-L_{n}\;W_{i}\right)/t, \end{array}$$

  $$\begin{array}{c} FCR=Feed\;intake\;\left(g\right)/Weight\;gain\;\left(g\right), \end{array}$$

  $$\begin{array}{c} \text{Survival rate (SR)}\%=100\times \left(\text{initial shrimp number}-\text{dead shrimp number}\right)/\left(\text{initial shrimp number}\right), \end{array}$$

where *W*_i_ is the initial body weight; *W*_f_ is the final body weight; *t* is the number of experimental days; *L*_f_ is the final length, and *L*_i_ is the initial length.

### 2.4. Immune-Antioxidant and Biochemical Parameters of Hemolymph

After 60 days, hemolymph samples were collected from three specimens per tank, as previously described [30, 31]. The samples were centrifuged at 6000 *g* for 10 min at 4°C to separate plasma from hemocytes. Biochemical and antioxidant parameters were measured using standard methods [27]. Catalase (CAT), glutathione peroxidase (GPX), superoxide dismutase (SOD), glucose, and total protein levels were determined using diagnostic reagent kits (Pars Azmon, Iran). Lysozyme activity was assessed using the turbidimetric method. Cholesterol levels were measured with an Autoanalyzer (Caretium 910362/XI 921A, Korea) and a Pars Azmoon diagnostic kit. Total hemocyte count (THC) was determined using a hemocytometer (×400). Differential hemocyte counts, including granular cells (GCs), semi-granular cells (SGCs), and hyaline cells (HCs), were performed by preparing and drying hemolymph, fixing it in methanol, staining with the May–Grunwald–Giemsa method, and counting under a light microscope.

### 2.5. Immune-Related Gene Expression

For RNA extraction, the hepatopancreas tissue from three individuals of each tank was sampled and preserved in RNA later. The extraction of RNA was carried out using 50–100 mg of tissue in the column kit from Dana Zist Asia (Dana Zist, Iran) according to the suggested protocol with some minor modifications. The extracted RNA was treated with DNase I (Fermentase, USA) in accordance with the manufacturers' instructions. RNA quantification was carried out with a NanoDrop ND-1000 Spectrophotometer (Thermo Scientific, Wilmington, DE, USA) reading at 260/280 nm and the quality of the RNA was measured by electrophoresis on a 1% agarose gel. One microgram of total RNA was used to synthesize first-strand cDNAs using ExcelRTReverse Transcriptase Kit (SMOBio, Taiwan) following the manufacturer's instructions. The transcripts of five immune-related genes, including *α2-M*, *integrin β*, *PX*, *LGBP*, and *PO1*, and the internal reference gene, actin beta (ACTB), were measured by quantitative real-time PCR on a Rotor-Gene Q Real Time PCR (Qiagene, Germany) using 2 μL of cDNA as a template, 0.5 μL of primers and 7.5 μL of Taq SYBR green (Ampliqon 2x SYBR green Low rox). Each qPCR experiment was performed on five shrimp per treatment in triplicates using a program of 95°C for 10 min; 40 cycles of denaturation at 94°C for 15 s, annealing at 59°C for 40 s and extension at 72°C for 20 s. The control samples were chosen as the reference calibrator samples for normalization of the mRNA expression of target genes. The sequences of the primers that were used for the qRT-PCR are presented in Supporting Information: 1 Table S1.

### 2.6. Statistical Analysis

All data were presented as means ± standard deviation (SD). Prior to statistical analysis, the normality of the data was assessed using the Shapiro–Wilk test. The dataset was then subjected to one-way ANOVA, followed by multiple comparisons utilizing Tukey's post hoc test for significant effects at a significance level of (*p* < 0.05). All statistical analyses were conducted using the SPSS program (version 16), and the data were visualized using SigmaPlot (version 11).

## 3. Results

### 3.1. Growth Parameters

Table 1 presents the results of growth parameters and survival of shrimp fed with different doses of LPE. Shrimp fed diets containing 1% LPE showed significantly higher WG, SGR, and TLI compared to the control group (*p* < 0.05). The feed conversion ratio (FCR) significantly decreased in the treatment fed with 1% LPE diet compared to the control group (*p* < 0.05). The survival rate in all control and LPE-fed treatments was above 91%, with no significant differences observed among the treatments.

### 3.2. Hemolymph Immune-Antioxidant and Biochemical Parameters

**Figure 1:**
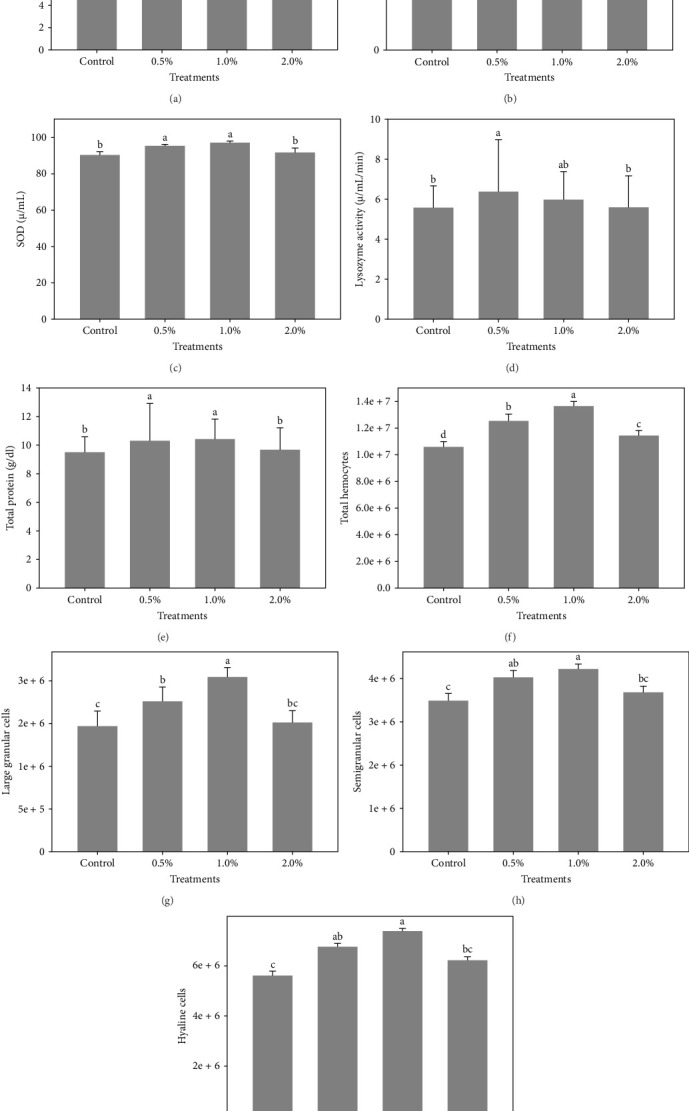
The results of immune-antioxidant biomarkers of hemolymph of shrimp fed diets containing 0.5%, 1.0%, and 2% ethanolic extract of lemon peel after 60 days of feeding trial. Data (mean ± SD) with different letters are significantly different among treatments according to the ANOVA and Turkey's post hoc test (*p* < 0.05). (A) CAT (*µ*/mL); (B) GPX (*µ* /mL); (C) SOD (*µ* /mL); (D) Lysozyme activity (*µ* /mL/min); (E) Total protein (g/dL); (F) Total hemocytes; (G) Large granular cells; (H) Semigranular cells; (I) Hyaline cells.

Figure 1 shows the results of immune-antioxidant parameters of shrimp fed with different doses of lemon peel. The activity of antioxidant enzymes, GPX (Figure 1B) and SOD (Figure 1C) and the levels of plasma protein (Figure 1E) were significantly higher in shrimp fed diets containing 0.5% and 1% LPE compared to the control group (*p* < 0.05). Moreover, the activities of CAT (Figure 1A) and lysozyme were significantly higher in shrimp fed diet containing 0.5% LPE compared to the control group (*p* < 0.05). Shrimp fed diets containing all three doses of LPE showed significantly higher counts of total hematocytes (Figure 1F) compared to the control group (*p* < 0.05). Furthermore, in shrimp fed diets containing 0.5% and 1% LPE, the counts of large granulocytes (Figure 1G), semi-granulocytes (Figure 1H), and hyaline cells (Figure 1I) were significantly increased compared to the control group (*p* < 0.05).

**Figure 2:**
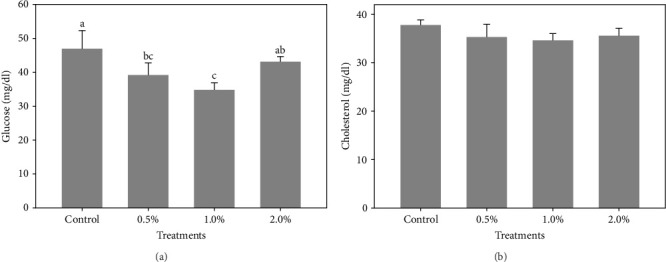
The glucose (A) and cholesterol (B) levels of hemolymph in *L. vannamei* fed diets containing 0.5%, 1.0%, and 2% ethanolic extract of lemon peel after 60 days of feeding trial. Data (mean ± SD) with different letters are significantly different among treatments according to the ANOVA and Turkey's post hoc test (*p* < 0.05).

The results also showed that the plasma glucose (Figure 2A) was significantly decreased in shrimp fed diets containing 0.5% and 1% LPE compared to the control group (*p* < 0.05). The cholesterol levels (Figure 2B) in shrimp fed with different doses of LPE did not show significant difference compared to the control treatment (*p* > 0.05); however, shrimp fed diets containing 0.5% and 1% LPE showed lower levels of plasma cholesterol compared to the control.

### 3.3. Immune-Related Gene Expression

**Figure 3:**
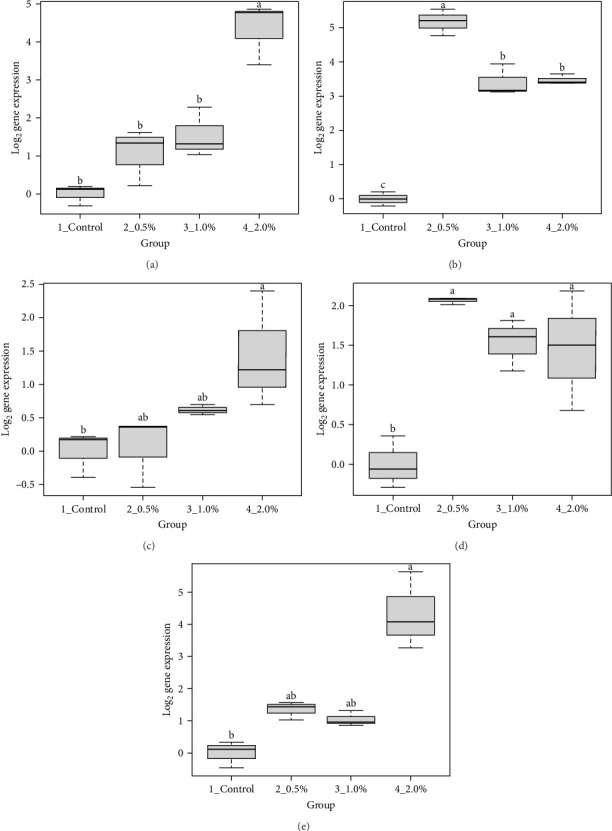
Log_2_ expression of (A) integrin β, (B) LGBP, (C) Peroxinectin (D) prophenoloxidase 1, and (E) α2-macroglobulin (α2-M) in hepatopancreas of *L. vannamei* fed diets containing 0.5%, 1.0%, and 2% ethanolic extract of lemon peel after 60 days of feeding trial. Data (mean ± SD) with different letters are significantly different among treatments according to the ANOVA and Turkey's post hoc test (*p* < 0.05).

The results showed that the transcripts of *integrin β* (Figure 3A), *PX* (Figure 3C), and *α2-M* (Figure 3E) were significantly upregulated in *L. vannamei* fed diets with 2% LPE compared to the control group (*p* < 0.05). The mRNA expression of *LGBP* (Figure 3B) and *proPO1* (Figure 3D) was significantly higher in shrimp fed with different doses of LPE compared to the control group (*p* < 0.05). The mRNA expression of *LGBP* in shrimp fed diet with 0.5% of LPE was also significantly higher than shrimp fed diets with 1% and 2% of LPE (*p* < 0.05).

## 4. Discussion

Citrus fruits, particularly lemons (*C. limon*), are rich in bioactive compounds such as polyphenolic flavonoids, vitamin C, and essential oils, which are known to exhibit antioxidant and anti-inflammatory properties [32, 33]. Studies have demonstrated that the incorporation of lemon peel in animal diets can enhance growth performance and boost immune function, likely due to the synergistic effects of its phytochemicals [34]. Therefore, in the present study, we supplemented the diet of cultured shrimp *L. vannamei* with different doses of LPE and investigated their effects on growth parameters, plasma immune-antioxidant levels, and the expression of hepatopancreatic immune-related genes. Supplementation of 1% LPE enhanced the growth performance of the shrimp. Moreover, most of the immune-antioxidant parameters of shrimp were significantly improved in shrimp fed with 0.5% and 1% LPE. The expression of immune-related biomarkers was also increased in shrimp fed with different doses of LPE.

In the present study, the incorporation of 1% *LPE* into shrimp diet demonstrated enhanced growth performance, including increased WG, SGR, and improved feed conversion ratio. Bioactive compounds in lemon peel, such as flavonoids and limonoids, are thought to enhance nutrient absorption and metabolic efficiency [34]. These compounds may also stimulate digestive enzymes and promote gut health, both of which are crucial for optimal shrimp growth [34]. Furthermore, the polyphenolic flavonoids found in lemon peel, such as hesperidin, quercetin, and rutin, have been shown to stimulate feeding behavior and promote growth [29]. Previous studies on various fish and shrimp species have shown mixed results, with growth depression observed in gilthead seabream, *S. aurata* [35], African catfish, *C. gariepinus* [21], Nile tilapia *O. niloticus*, and rainbow trout, *O. mykiss* when fed 1%–3% dried lemon peel. In contrast, studies on Asian sea bass, *L. calcarifer* [23], orange-spotted grouper, *E. coioides* [24], rohu carp, *Labeo rohita* [29] and white Pacific shrimp, *L. vannamei* [25] have shown no negative effects or positive effects on growth performance when up to 3% of the diet consists of fermented or extract of lemon peel with. These findings, together with the results of the present study, suggest that LPE may be a promising additive for enhancing growth in aquaculture species.

In addition to its growth-promoting effects, LPE has been associated with improved plasma antioxidant biomarkers in shrimp. Antioxidants are critical for protecting organisms from oxidative stress, which can be exacerbated by environmental factors and pathogens. Our results indicate that dietary LPE, at levels up to 1%, enhances the antioxidant capacity of shrimp. This improvement in antioxidant enzyme activity may contribute to better overall health in shrimp. Strengthening antioxidant defenses is vital for maintaining immune function and resilience against diseases, which are common challenges in shrimp aquaculture. SOD, GPx, and CAT are well-established as the first line of defense against oxidative stress in fish and shrimp, and their activation helps neutralize the harmful effects of reactive oxygen species (ROS) [36]. The bioactive compounds in LPE, such as hesperidin, are likely responsible for this enhancement. Hesperidin may reduce lipid peroxidation by binding hydrogen ions to free radicals, a mechanism similar to that of other antioxidants like vitamins E and C [35]. Previous studies have demonstrated the beneficial effects of lemon peel on the antioxidant systems of various fish species and shrimp. For instance, supplementation with dried lemon peel increased the activity of SOD, CAT, and GPx in rohu carp, *L. rohita* [36], while dehydrated lemon peel improved antioxidant enzyme activity in both Nile tilapia, *O. niloticus*, African catfish, *C. gariepinus* [21], and in rainbow trout, *O. mykiss*) under crowding stress [28]. More recent research on shrimp showed that feeding with 2% fermented lemon peel led to a significant increase in phenoloxidase (PO) activity compared to the control group [25]. Collectively, the enhanced antioxidant enzyme activity observed in this and previous studies further supports the positive effects of lemon peel on the antioxidant system and its protective role against oxidative damage in shrimp.

Our results also highlight the beneficial role of LPE in enhancing certain immune biomarkers in the hemolymph of shrimp, including lysozyme activity, total protein levels, and hematological indices. Lysozyme, a crucial component of innate immunity, plays a key role in defending against pathogens and oxidative stress, particularly in species that lack an adaptive immune system [23, 24, 37]. Shrimp fed a diet supplemented with 0.5% LPE exhibited the highest plasma lysozyme activity. Similar effects have been observed in fish species, where dietary lemon peel supplementation increased plasma lysozyme activity. For instance, dried and fermented lemon peel boosted lysozyme activity in the orange-spotted grouper, *E coioides* [24] and in rohu carp, *L. rohita* [36]. Additionally, in line with studies on fish [36], LPE supplementation also elevated plasma total protein levels in shrimp. In penaeid shrimp, circulating hemocytes are vital to the non-specific immune defense system [25, 38]. Our findings demonstrated significantly higher hematological indices, including total hemocytes, large granulocytes, semi-granulocytes, and hyaline cells, in shrimp fed diets supplemented with LPE compared to the control group. The production of hemocytes under normal, non-infectious conditions is essential for immunity, and hemocyte counts are an effective method for assessing the immune status of shrimp [30, 39]. Hemocytes, which perform multiple roles in innate immunity such as encapsulation, coagulation, melanization, and phagocytosis, are critical for immune responses in crustaceans [40]. Hemocyte counts are commonly used to evaluate the immunity of *L. vannamei* [40–42]. Moreover, prior research has demonstrated the positive impact of diets containing fermented lemon on hemocyte counts in both fish [23, 24] and shrimp [25]. Studies on various fish species also suggest that dried lemon peel supplementation can enhance immune function [20, 21, 28]. The immune-stimulatory effects of lemon peel are likely attributed to its bioactive compounds [43]. While this study did not specifically identify the phytochemicals or metabolites responsible for the observed immune effects in shrimp, it clearly demonstrates that LPE supplementation confers health benefits for shrimp [25].

The results of this study also demonstrated a reduction in hemolymph glucose and cholesterol levels in shrimp fed diets supplemented with LPE. Citrus peels, including those from orange, grapefruit, and lemon, are known for their hypoglycemic effects [44]. The hypoglycemic activity of citrus peels is attributed to bioactive compounds such as hesperidin (a glycosylated flavanone derived from hesperetin), α-pinene, and β-pinene [45, 46]. Similar findings have been reported in fish species, where dietary supplementation with lemon peel reduced serum glucose levels in gilthead seabream, *S. aurata* [20], Nile tilapia, *O. niloticus* [21], and tilapia, *O. mossambicus* fed with orange peel extract [46]. The hypocholesterolemic effect of lemon peel is also well established, with studies indicating that pectin, a soluble fiber in lemon peels, can reduce cholesterol by promoting the excretion of bile acids, which bind to cholesterol [47, 48]. Consistent with this, dietary dehydrated lemon peel has been shown to reduce plasma cholesterol levels in gilthead seabream, *S*. *aurata* [20], and orange peel extract has had a similar effect on plasma cholesterol in tilapia, *O*. *mossambicus* [46]. These findings support the potential of LPE as a dietary supplement for modulating glucose and cholesterol levels in shrimp.

In this study, we assessed the mRNA expression of a panel of immune-related genes in shrimp following dietary supplementation with LPE. Our findings revealed a significant upregulation of immune-related genes in shrimp fed diets containing varying doses of LPE. Notably, the expression of *LGBP* and *proPO1* was markedly increased in response to all tested doses of LPE. Melanization, a key immune response in penaeid shrimp, is facilitated by PO and regulated through the proPO activation cascade [49]. This cascade is triggered by the binding of pattern-recognition proteins to pathogen-associated molecular patterns (PAMPs). LGBP, a pattern-recognition protein, recognizes lipopolysaccharides and β-1,3-glucans found in pathogen cell wall components [50, 51]. PO subsequently oxidizes phenolic compounds into quinones, which polymerize to form melanin [52]. Given that LPE is rich in phenolic compounds such as phenolic acids, flavanones, and polymethoxylated flavones [53], it may enhance proPO activity in the shrimp hepatopancreas, a critical organ in immune regulation. Previous studies in fish have also shown that dietary lemon peel supplementation can upregulate the expression of immune-related genes. For example, in rohu carp, *L. rohita*, dried lemon peel supplementation enhanced immune gene expression [36], and in gilthead seabream, *S. aurata*, dehydrated lemon peel stimulated immune-related gene expression [20]. The modulation of immune-related genes by LPE in shrimp highlights its potential as a natural immunostimulant in aquaculture. This enhanced gene expression reflects an activated immune system, potentially improving shrimp resilience to pathogenic challenges.

## 5. Conclusion

In this study, we investigated the effects of LPE inclusion on growth parameters, hemolymph immune-antioxidant and biochemical profiles, as well as hepatopancreatic immune-related gene expression in shrimp. Our results indicate that incorporating LPE into shrimp diets, particularly at 0.5% and 1%, can enhance growth performance and strengthen immune-antioxidant function, likely due to the synergistic effects of its phytochemicals and bioactive components. These findings highlight the potential of LPE not only to support growth but also to play a vital role in enhancing the immune system of shrimp. Therefore, LPE presents a promising, cost-effective feed additive for shrimp aquaculture, with the potential to improve disease resistance and overall health.

## Figures and Tables

**Table 1:** Growth parameters and survival of *L. vannamei* fed diets containing 0.5%, 1.0%, and 2% ethanolic extract of lemon peel after 60 days of feeding trial.

Items	Treatment			
	0.0% LPE (control)	0.5% LPE	1% LPE	2% LPE
*W*_i_ (g)	4.33 ± 0.47	4.10 ± 0.30	4.17 ± 0.12	4.17 ± 0.40
*W*_f_ (g)	11.07 ± 0.71^b^	11.80 ± 0.36^b^	14.77 ± 0.68^a^	11.60 ± 0.85^b^
WG (g)	6.73 ± 0.25^b^	7.70 ± 0.52^b^	10.6 ± 0.60^a^	7.43 ± 0.64^b^
*L*_i_ (mm)	78.5 ± 5.1	74.7 ± 3.3	73.7 ± 1.3	75.3 ± 2.5
TLI (mm)	28.3 ± 2.8^c^	35.0 ± 3.0^b^	43.0 ± 2.9^a^	33.4 ± 2.6^bc^
SGR	1.59 ± 0.08^b^	1.79 ± 0.14^b^	2.14 ± 0.05^a^	1.73 ± 0.12^b^
FCR	1.70 ± 0.06^ab^	1.53 ± 0.10^b^	1.29 ± 0.07^c^	1.89 ± 0.17^a^
SR (%)	96.7 ± 3.5	96.0 ± 2.0	91.3 ± 1.2	94.0 ± 2.0

*Note*: Data (mean ± SD) with different letters are significantly different among treatments according to ANOVA test (*p* < 0.05).

Abbreviations: FCR, feed conversion ratio; *L*_i_, initial length; SGR, specific growth rate; SR, survival rate; TLI, total length increase; *W*_f_, final weight; WG, weight gain; *W*_i_, initial weight.

## Data Availability

The data that support the findings of this study are available from the corresponding author upon reasonable request.

## Supporting Information

Supporting Information Table S1. Details of the primer pairs used for gene expression analysis in *L. vannamei* in this study.
