# Supplementary material for: Dietary Supplementation of Ethanolic Lemon Peel (Citrus limon) Extract Enhances Growth Performance and Immune-Antioxidant Responses in Pacific White Shrimp (Litopenaeus vannamei)
Source: Aquac Nutr. 2025 Jun 30;2025:4879504. doi: 10.1155/anu/4879504 (PMC12234166; doi:10.1155/anu/4879504)
Supplement: Supporting Information — Table S1. Details of the primer pairs used for gene expression analysis in L. vannamei in this study. [file 4879504.f1.docx]

Supplementary Table 1. Details of the primer pairs used for gene expression analysis in *L. vannamei* in this study.

| **Primer name** | **Sequence** | **TM (°C)°** | **Amplicon** |
| --- | --- | --- | --- |
| ***integrin β*** | F-ATGCCAGGAGACACTTACGC  R-GCCCAAAACACTCCTTGCTG | 60  60 | 148 |
| **Peroxinectin** | F-TCTCCCCATCTCATCCAGCA  R-CCGAAGTTTACTCTGGGCGT | 60  60 | 140 |
| ***α2-macroglobulin* (*α2-M*)** | F-GATCAACTTCGCCCCAAACG R-GAAGGCGCTGTAGGAACCAT | 60  60 | 154 |
| ***beta-1,3-glucan-binding protein* (LGIP)** | F-ATGTCTGGGAGCACGAGTTG R-AGTCATCGCCCTTCCAGTTG | 60  60 | 144 |
| ***Prophenoloxidase* 1 (*PO1*)** | F-ACGGCTACTTCCCCAAACTG  R-CGCCAGATTTCCAGGTCAGT | 60  60 | 136 |
| **Actin beta (ACTB)** | F-GCTCGTCCGCCCTTTTAGTA R-GATGGAGGGGAACACAGCTC | 60 60 | 141 |
